# Urticaria multiforme: a case report in an infant^☆^^☆☆^

**DOI:** 10.1016/j.abd.2019.09.005

**Published:** 2019-09-30

**Authors:** Maria Claudia Alves Luce, Bruno de Castro e Souza, Maria Fernanda Vieira Cunha Camargo, Neusa Yuriko Sakai Valente

**Affiliations:** a Department of Dermatology, Hospital do Servidor Estadual de São Paulo, São Paulo, SP, Brazil; b Department of Pediatric Dermatology, Hospital do Servidor Público Estadual de São Paulo, São Paulo, SP, Brazil; c Department of Dermatopathology, Hospital do Servidor Público Estadual de São Paulo, São Paulo, SP, Brazil

Dear Editor,

Urticaria multiforme (UM) is an uncommon benign cutaneous hypersensitivity that occurs mainly in pediatric patients.^1^, ^2^ It is characterized by annular lesions with a violaceous center, and may be accompanied by short-term fever, as well as by hand and foot edema.^2^, ^3^ It is a poorly recognized condition, mainly due to lack of reports in the literature. Furthermore, it is an important differential diagnosis for erythema multiforme.

A female infant was born at 31 weeks due to intrauterine growth restriction. After receiving the meningococcal and pneumococcal vaccines when she was 4 months old, annular macules with erythematous borders and red-frosted centers appeared (Fig. 1). The lesions presented an ephemeral character (24 h), with new macules appearing concomitantly. Due to age, it was not possible to evaluate pruritus. The diagnostic hypotheses were urticaria multiforme and childhood annular erythema. A biopsy was conducted, revealing preserved epidermis, a superficial and deep perivascular and interstitial inflammatory lymphohistiocytic infiltrate permeated with some eosinophils. There was no sign of vasculitis, corroborating with the diagnosis of urticaria multiforme (Figure 2, Figure 3). Antihistamine treatment (hydroxyzine 0.5 mg/kg every 12 h) was started. After ten days, the condition resolved completely, without residual lesions.

**Figure 1:**
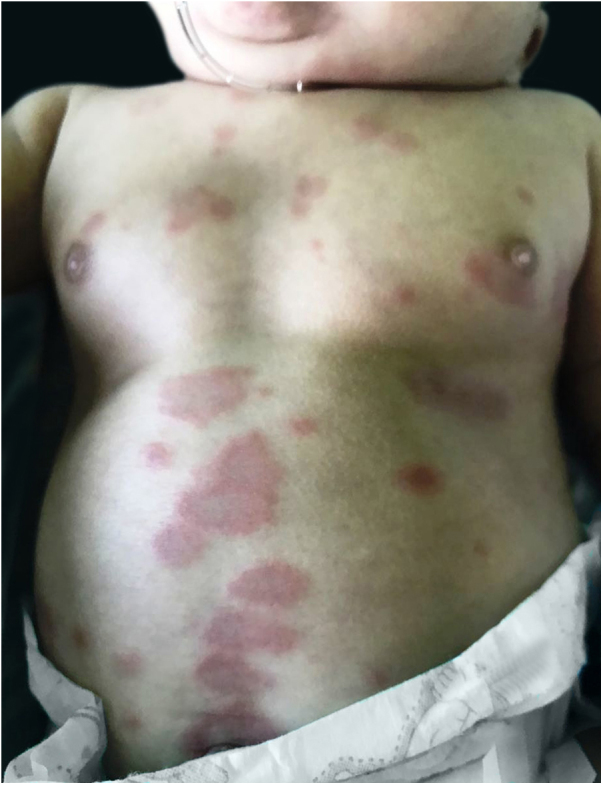
Erythematous annular plaques with lighter centers in the abdomen.

**Figure 2:**
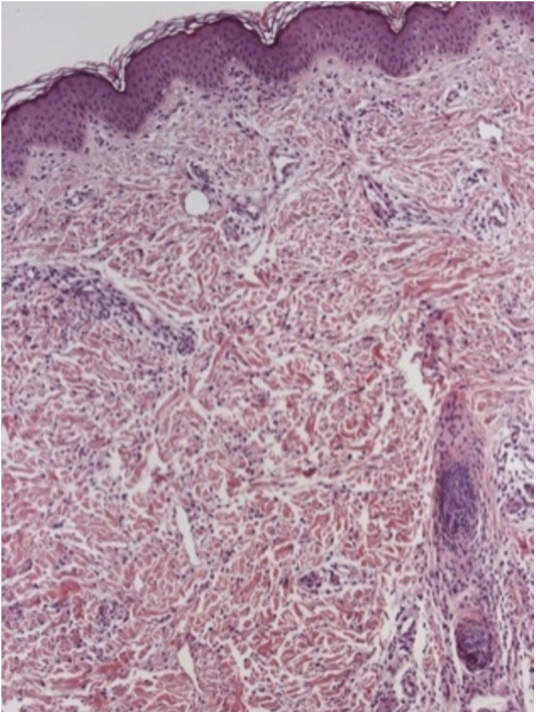
Inflammatory lymphocytic and histiocytic perivascular and interstitial infiltrate permeated with some eosinophils. Hematoxylin & eosin, ×100.

**Figure 3:**
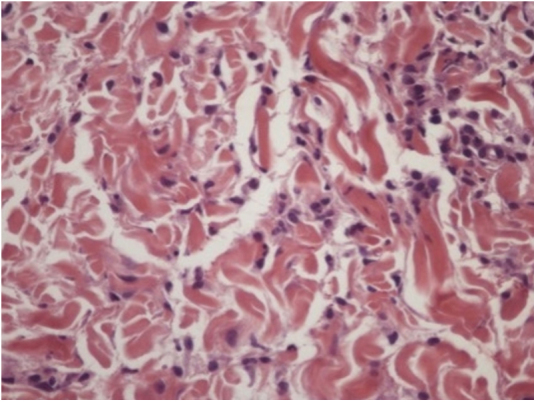
Interstitial histiocytic infiltrate with eosinophils. Hematoxylin & eosin, ×400.

Originally described in 1997 by Tamayo-Sanchez et al. under the name of acute annular urticaria,^1^ it had its name changed to urticaria multiforme in 2007 due to clinical similarity with erythema multiforme,^2^ as described by Shah et al., when the authors reported 19 of these cases. They also proposed that this condition is a variant of common urticaria, since the patients presented pruritus and dermographism.^1^, ^2^

With few cases in the literature, the etiology is poorly understood. In most cases there is a temporal relationship with infections (mycoplasma, adenovirus, streptococci, Epstein–Barr), medications (mainly antibiotics), and vaccinations. In 2016, Sempau et al. found an association with previous use of amoxicillin.^3^ Specifically in neonates, there has been proven infection by herpes virus 6, and decreased viral load accompanied by clinical improvement of the patient.^4^

The most affected age range is between 4 months and 4 years, although newborns and adolescents may also develop the condition. Cutaneous lesions begin as urticarial lesions that rapidly expand in a centrifugal manner and become annular, forming coalescent polycyclic plaques with violet or opaque red centers. Individually, each lesion dissipates within 24 h. Edema of the hands, feet, and face are important clinical signs, present in 61% of cases.^2^ The most present symptom is pruritus (94%); nonetheless, it can be difficult to evaluate in many young patients.^2^ Fever and dermographism are present in 44% of the cases; however, the general condition is preserved.^2^ The lesions resolve spontaneously within ten days without scars.

The diagnostic criteria are: annular, transitory, ecchymotic-center plaques, each lesion lasting less than 24 h, associated fever episode, total duration of the condition being less than ten days, and edema of the extremities.^3^ Laboratory tests are not necessary, a good anamnesis and a dermatological physical examination are sufficient.

As in most cases the diagnosis is made clinically, the histopathological records are quite scarce. Findings of superficial dermal edema associated with perivascular and interstitial lymphocytic infiltrates with eosinophils and occasionally neutrophils predominate. In one of the cases reported by Samorano et al. there was presence of histiocytes, as well as in the report presented here, but this is not a common finding.^5^

The main differential diagnosis is erythema multiforme. Initially, many patients are mistakenly diagnosed with erythema multiforme and subsequently the diagnosis is corrected. Important clinical findings for differentiation are the rapid resolution of urticarial multiforme lesions (<24 h), and the presence of a necrotic center, which is present in erythema multiforme. Another differential diagnosis is acute urticaria, in which there is intense pruritus, but no fever, and the lesions do not have an equinox center.^2^ In children with fever and acral face edema, it must be differentiated from a reaction similar to serum sickness, which occurs after administration of animal serum or foreign proteins. The most common clinical condition includes fever, arthralgia, angioedema, urticaria, and lymphadenopathy.^2^, ^3^ It can also present annular centrifugal erythema, migratory chronic erythema, viral rash, urticaria vasculitis, other vasculitis, and lupus erythematosus.

The treatment of urticaria multiforme is symptomatic, since there is spontaneous resolution in most cases, as seen in the patient of this report. Any suspected and unnecessary medications should be discontinued.^1^ Systemic antihistamines should be prescribed to relieve symptoms. In refractory and severe cases, the use of systemic corticosteroids in combination with antihistamines may be necessary.^2^, ^3^, ^5^

## Author's contribution

Maria Claudia Alves Luce: Conception and planning of the study; elaboration and writing of the manuscript; critical review of the literature.

Bruno de Castro e Souza: Conception and planning of the study; elaboration and writing of the manuscript; critical review of the literature.

Maria Fernanda Vieira Cunha Camargon: Approval of the final version of the manuscript; intellectual participation in propaedeutic and/or therapeutic conduct of the cases studied; critical review of the manuscript.

Neusa Yuriko Sakai Valente: Approval of the final version of the manuscript; intellectual participation in propaedeutic and/or therapeutic conduct of the cases studied; critical review of the manuscript.

## Financial support

None declared.

## Conflicts of interest

None declared.
